# Supplementary material for: Exploration of the sensitivity to macrocyclic lactones in the canine heartworm (Dirofilaria immitis) in Australia using phenotypic and genotypic approaches
Source: Int J Parasitol Drugs Drug Resist. 2022 Nov 15;20:145–58. doi: 10.1016/j.ijpddr.2022.11.003 (PMC9772245; doi:10.1016/j.ijpddr.2022.11.003)
Supplement: Multimedia component 1 [file mmc1.docx]

**Supplementary Table S1:** Primers used throughout the study

| **Assay specificity** | **Application** | **Target** | **Primer/probe** | **ID** | **Sequence (5'-3')** | **Reference** |
| --- | --- | --- | --- | --- | --- | --- |
| **Dog** | qPCR | ***GAPDH*** | Primer | S0631_DOG_F, S1072_DOG_F | TCAACGGATTTGGCCGTATTGG | (Orr et al., 2020; Panetta et al., 2021) |
|  |  |  |  | S0634_DOG_R, S1073_DOG_R | TGAAGGGGTCATTGATGGCG |  |
|  |  |  | Probe | S0632_DOG_P, S1074_DOG_P | CAGGGCTGCTTTTAACTCTGGCAAAGTGGA |  |
| ***Dirofilaria immitis*** | qPCR | ***cox1*** | Primer | S0624_F | TAGAGGGTCAGCCTGAGTTATC | (Panetta et al., 2021) |
|  |  |  |  | S0626_R | AGTAGAACGTATATTCTGAACAGTAACC |  |
|  |  |  | Probe | S0625_P | AGAACCAATACCAACAGTATGAAGACC |  |
| ***Dirofilaria immitis*-associated *Wolbachia*** | qPCR | ***ftsZ*** | Primer | WDiro.ftsZ.490-F | AAGCCATTTRGCTTYGAAGGTG | (Laidoudi et al., 2020) |
|  |  |  |  | WDiro.ftsZ.600-R | AAACAAGTTTTGRTTTGGAATAACAAT |  |
|  |  |  | Probe | WDimm.ftsZ.523-P | CGTATTGCAGAGCTCGGATTA |  |
| ***Dirofilaria immitis* L42411 SNP** | Illumina amplicon NGS | **L42411** | Primer with Illumina overhang adapter | S1020_NODE_42411_FOR | TCGTCGGCAGCGTCAGATGTGTATAAGAGACAGTTCTATCGAAAACCTTCCAG | (Bourguinat et al., 2015) |
|  |  |  |  | S1021_NODE_42411_REV | GTCTCGTGGGCTCGGAGATGTGTATAAGAGACAGAGGTTGCAAAAGTTGCAATG |  |
| ***Dirofilaria immitis* L21554 SNP** | Illumina amplicon NGS | **L21554** | Primer with Illumina overhang adapter | S1012_NODE_21554_FOR | TCGTCGGCAGCGTCAGATGTGTATAAGAGACAGCATCGTTGTCAACTTCCTGC | (Bourguinat et al., 2015) |
|  |  |  |  | S1013_NODE_21554_REV | GTCTCGTGGGCTCGGAGATGTGTATAAGAGACAGGAAATTTGAAAATGGGTACT |  |
| ***Dirofilaria immitis* L45689 SNP** | Illumina amplicon NGS | **L45689** | Primer with Illumina overhang adapter | S1022_NODE_45689_FOR | TCGTCGGCAGCGTCAGATGTGTATAAGAGACAGACGCAGGAAAGCTTTAATGG | (Bourguinat et al., 2015) |
|  |  |  |  | S1023_NODE_45689_REV | GTCTCGTGGGCTCGGAGATGTGTATAAGAGACAGATCATCATTTTATCAATTCC |  |
| ***Dirofilaria immitis* L9400 SNP** | Illumina amplicon NGS | **L9400** | Primer with Illumina overhang adapter | S1026_NODE_9400_FOR | TCGTCGGCAGCGTCAGATGTGTATAAGAGACAGGTTATTTGCACTACTCTCCC | (Bourguinat et al., 2015) |
|  |  |  |  | S1027_NODE_9400_REV | GTCTCGTGGGCTCGGAGATGTGTATAAGAGACAGTGGCGTACTGATCACATTGG |  |
| ***Dirofilaria immitis* L20587 SNP** | Illumina amplicon NGS | **L20587** | Primer with Illumina overhang adapter | S1010_NODE_20587_FOR | TCGTCGGCAGCGTCAGATGTGTATAAGAGACAGTCGATCATTTAGTAACAACG | (Bourguinat et al., 2015) |
|  |  |  |  | S1011_NODE_20587_REV | GTCTCGTGGGCTCGGAGATGTGTATAAGAGACAGTTGCGTTACAGCGCCAAATC |  |
| ***Dirofilaria immitis* L15709A SNP** | Illumina amplicon NGS | **L15709A** | Primer with Illumina overhang adapter | S1008_NODE_15709_A_FOR | TCGTCGGCAGCGTCAGATGTGTATAAGAGACAGGGCCAATAAATAAAGGCTA | (Bourguinat et al., 2015) |
|  |  |  |  | S1009_NODE_15709_A_REV | GTCTCGTGGGCTCGGAGATGTGTATAAGAGACAGGTTTTCTGGAATTATCAGAC |  |
| ***Dirofilaria immitis* L30575 SNP** | Illumina amplicon NGS | **L30575** | Primer with Illumina overhang adapter | S1018_NODE_30575_FOR | TCGTCGGCAGCGTCAGATGTGTATAAGAGACAGCGAGGTAAAGCACACAGAAG | (Bourguinat et al., 2015) |
|  |  |  |  | S1019_NODE_30575_REV | GTCTCGTGGGCTCGGAGATGTGTATAAGAGACAGCAACAAAATGCCGCAGATGG |  |

**References:**

Bourguinat, C., Lee, A.C., Lizundia, R., Blagburn, B.L., Liotta, J.L., Kraus, M.S., Keller, K., Epe, C., Letourneau, L., Kleinman, C.L., Paterson, T., Gomez, E.C., Montoya-Alonso, J.A., Smith, H., Bhan, A., Peregrine, A.S., Carmichael, J., Drake, J., Schenker, R., Kaminsky, R., Bowman, D.D., Geary, T.G., Prichard, R.K., 2015. Macrocyclic lactone resistance in *Dirofilaria immitis*: Failure of heartworm preventives and investigation of genetic markers for resistance. Vet Parasitol 210, 167-178. doi: 10.1016/j.vetpar.2015.04.002

Laidoudi, Y., Davoust, B., Varloud, M., Niang, E.H.A., Fenollar, F., Mediannikov, O., 2020. Development of a multiplex qPCR-based approach for the diagnosis of *Dirofilaria immitis*, *D. repens* and *Acanthocheilonema reconditum*. Parasit Vectors 13, 319. doi: 10.1186/s13071-020-04185-0

Orr, B., Ma, G., Koh, W.L., Malik, R., Norris, J.M., Westman, M.E., Wigney, D., Brown, G., Ward, M.P., Šlapeta, J., 2020. Pig-hunting dogs are an at-risk population for canine heartworm (*Dirofilaria immitis*) infection in eastern Australia. Parasit Vectors 13, 69. doi: 10.1186/s13071-020-3943-4

Panetta, J.L., Calvani, N.E.D., Orr, B., Nicoletti, A.G., Ward, M.P., Šlapeta, J., 2021. Multiple diagnostic tests demonstrate an increased risk of canine heartworm disease in northern Queensland, Australia. Parasites & vectors 14, 393-393. doi: 10.1186/s13071-021-04896-y
