# Supplementary material for: Exploration of the sensitivity to macrocyclic lactones in the canine heartworm (Dirofilaria immitis) in Australia using phenotypic and genotypic approaches
Source: Int J Parasitol Drugs Drug Resist. 2022 Nov 15;20:145–58. doi: 10.1016/j.ijpddr.2022.11.003 (PMC9772245; doi:10.1016/j.ijpddr.2022.11.003)
Supplement: Multimedia component 2 [file mmc2.docx]

**Supplementary Table S2:** SNPs in this study adopted from previous 5-SNPs models from the USA

| **SNP position** | **Node / locus** | **SNP included in 5-SNPs model** | | **SNP included** |
| --- | --- | --- | --- | --- |
|  |  | (Ballesteros et al., 2018) | (Bourguinat et al., 2017) | **This study** |
| nDi.2.2.scaf00046:76278 | 15709_A | Yes | No | Yes |
| nDi.2.2.scaf00046:22857 | 30575 | Yes | No | Yes |
| nDi.2.2.scaf00046:222254 | 21554 | Yes | Yes | Yes |
| nDi.2.2.scaf00185:10639 | 45689 | Yes | Yes | Yes |
| nDi.2.2.scaf00185:62174 | 20587 | Yes | Yes | Yes |
| nDi.2.2.scaf00001-466197 | 42411 | No | Yes | Yes |
| nDi.2.2.scaf00005-662854 | 9400 | No | Yes | Yes |

**References**

Ballesteros, C., Pulaski, C.N., Bourguinat, C., Keller, K., Prichard, R.K., Geary, T.G., 2018. Clinical validation of molecular markers of macrocyclic lactone resistance in *Dirofilaria immitis*. International Journal for Parasitology: Drugs and Drug Resistance 8, 596-606. doi: 10.1016/j.ijpddr.2018.06.006

Bourguinat, C., Keller, K., Xia, J., Lepage, P., McTier, T.L., Woods, D.J., Prichard, R.K., 2017. Genetic profiles of ten *Dirofilaria immitis* isolates susceptible or resistant to macrocyclic lactone heartworm preventives. Parasites & vectors 10, 504-504. doi: 10.1186/s13071-017-2428-6
